# Surgical Neurology
*Abridged from an Address delivered to the Devon and Exeter Medico-Chirurgical Society on Thursday, 26th October, 1950.


**Published:** 1951-04

**Authors:** George L. Alexander

**Affiliations:** Neurological Surgeon, United Bristol Hospitals and South-Western Region.


### SURGICAL NEUROLOGY*

BY

GEORGE L. ALEXANDER, F.R.C.S.

*Neurological Surgeon, United Bristol Hospitals and South-Western Region.*

It is my intention to discuss some of the diagnostic considerations in the commoner conditions for which neurosurgical treatment is indicated, with special reference to choice of patients for specialist consultation. At the outset, I wish to emphasize the importance to the surgeon nowadays of forecasting in advance if possible the pathology of the lesion to be treated, because the plan of attack sometimes depends on fore-knowledge of the pathology.

(Operations for alleviation of pain were touched on, with special reference to trigeminal neuralgia. The characteristics of the paroxysmal neuralgia were outlined and the importance of differentiating the true paroxysmal neuralgia from central trigeminal pain in advising treatment was emphasized.)

The neuralgia which can be cured by interruption of conduction in the trigeminal nerve comes in attacks, is frequently precipitated by stimuli on skin of the face of mucosa or mouth, is brief in duration and ceases *completely* between attacks. I believe that in alcohol injection, if the alcohol is injected slowly and in sufficient amount *into* the ganglion, the resulting anaesthesia and alleviation of pain are permanent. This procedure occupies about as much time as an operation. Operation is chosen for those cases in which the second and third division areas are affected by the pain: the virtue of operation is the preservation in most cases of corneal sensation. In dealing with the danger of trophic keratitis where the cornea is anaesthetic, avoid eye-drops and ointments, particularly for instillation by the patient. Another indication for root-section is found in some cases of trigeminal pain due to ineradicable peripheral foci of irritation.

* Abridged from an Address delivered to the Devon and Exeter Medico-Chirurgical Society on Thursday, 26th October, 1950.

Another field for surgery is found in intractable pain in the limbs and trunk. Cordotomy is not invariably successful; and in cases of painful stump and phantom limb, percussion of the stump, a treatment which incidentally originated from Dr. Carleton in Bristol, is now largely replacing antero-lateral cordotomy.

On the subject of epilepsy, two points are important: eye-witness accounts of attacks and the need for relatives to accompany patients at consultation. If epilepsy occurs for the first time in an adult there is a responsibility for the clinician to exclude tumour as a cause of the epilepsy. Encephalography or arteriography are often required in the investigation.

Spontaneous subarachnoid haemorrhage is commonly due to leakage from an intracranial aneurysm or congenital angioma: many of the patients are young persons. Recent advances in technique have greatly extended the scope and possibilities of surgery in the cure of those lesions.

Let us now turn our attention to conditions of the spine and spinal cord. Epidural spinal abscess is a surgical emergency requiring immediate treatment. The danger in that condition arises from the risk that clotting in epidural veins will become sufficiently extensive to arrest venous drainage from the cord, with softening of the cord as a result. This risk is real because the organism in epidural spinal abscess is so frequently a coagulase-positive staphylococcus. Let me remind you of the apyrexial character of some cases in this condition, and the frequency of root pains in most cases.

Another emergency situation, not yet widely recognized, is compression of the cauda equina by massive protrusion of lumbar disc. Persisting pressure on the sensory roots could cause permanent damage to them. I have encountered cases in which control of the sphincters, and potency, were permanently lost after a very short period of compression.

Head injuries: only a minority of cases require surgery. In closed head injury the most important guide in assessment is the " drift " of the case. Deterioration, under observation, in state of consciousness or in neurological findings is an indication for obtaining a neurosurgical opinion. In cases with rapid deterioration, notably where there were early signs of extradural haemorrhage, neurosurgical advice should be sought by telephone, and operation might best be undertaken on the spot if at a distance from the neurosurgical centre. Lives have been saved by co-operation and prompt action on those lines.

(Mr. Alexander also dealt briefly with infantile hydrocephalus and spina bifida, with brain abscess, and with essential and malignant hypertension, giving some hints as to selection of cases for surgery.)

The chief criterion in spina bifida is competency of the sphincters. I do not advise operation if the infant shows retention of urine with overflow and has a patulous anus: there is always a " saddle " anaesthesia in such cases. The caudal end of the cord in gross degrees of spina bifida is deficient and repair of the defect cannot make good that deficiency of neural tissue.

